# Supplementary material for: Transient Motion Classification Through Turbid Volumes via Parallelized Single-Photon Detection and Deep Contrastive Embedding
Source: Front Neurosci. 2022 Jul 8;16:908770. doi: 10.3389/fnins.2022.908770 (PMC9304989; doi:10.3389/fnins.2022.908770)
Supplement: Supplementary file 1 [file Image_1.pdf]

Transient motion classification through turbid volumes via parallelized single-photon detection and deep contrastive embedding: Supplementary Material

1 SYSTEM DESIGN

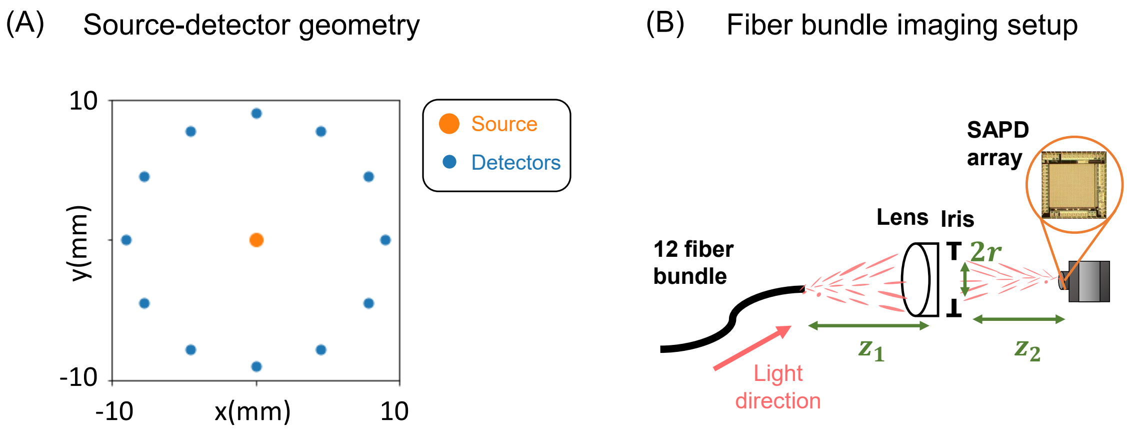

Figure S1. System design of *CREPE*. (A) depicts the source-detector geometry. 12 detector fibers are placed circularly around the center source. (B) An single-lens imaging setup was used to map the distal end of the fiber to the SPAD array. Assuming fully developed speckles are collected on the surface with an average speckle size of $\lambda/2$. To map one speckle on average to one SPAD pixel with size a with a fixed magnification $M = z_2/z_1$ using a single lens, we tune the iris radius to be $r = \lambda M/2\phi z_1$ to reduce the NA of the system.
